# Novel TLR7 gain-of-function variant and review of the associated disease spectrum

**DOI:** 10.70962/jhi.20250199

**Published:** 2026-01-12

**Authors:** Maud Tusseau, Claire Desvignes, Guilaine Boursier, Clémentine Fort, Clémence David, Marie-Louise Frémond, Sarah Benezech, Samira Khaldi-Plassart, Antonin Chenel, Jade Cognard, Liliane Khoryati, Jonathan Sormani, Frédérique Dijoud, David Goncalves, Anais Nombel, Maurine Jouret, Anne-Laure Mathieu, Alexandre Belot

**Affiliations:** 1 Inserm U1111, CNRS, UMR5308, ENS of Lyon, International Center of Infectiology Research, Claude Bernard University Lyon 1, Lyon, France; 2 Department of Medical Genetics, Hospices Civils de Lyon, University Hospital of Lyon, Lyon, France; 3 GCS AURAGEN, Lyon, France; 4 Pediatric Nephrology, Rheumatology, Dermatology Department, National Reference Centre on Rheumatic, Autoimmune Diseases in Children and Type I Interferonopathies, Hôpital Femme Mère Enfant, Hospices Civils de Lyon, University Hospital of Lyon, Lyon, France; 5 Department of Molecular Genetics and Cytogenomics, CHU Montpellier, IRMB U1183, University of Montpellier, Montpellier, France; 6 National Reference Centre for Autoinflammatory Diseases and Amyloidosis, Montpellier, France; 7 Pediatric Neurology, Hôpital Femme Mère Enfant, Hospices Civils de Lyon, University Hospital of Lyon, Lyon, France; 8 Laboratory of Neurogenetics and Neuroinflammation, Imagine Institute, Inserm UMR1163, Université Paris Cité, Paris, France; 9 Department of Internal Medicine, Hôpital Bichat-Claude Bernard, Assistance Publique Hôpitaux de Paris, Université Paris Cité, Paris, France; 10 Pediatric Immunohematology & Rheumatology Unit, National Reference Centre on Rheumatic, Autoimmune Diseases in Children and Type I Interferonopathies, Necker Hospital, Paris, France; 11 Pediatric Immunohematology and Oncology Institute, Hospices Civils de Lyon/Centre Léon Bérard, Lyon, France; 12 Pathology Department, Hospices Civils de Lyon, University Hospital of Lyon, Lyon, France; 13 Immunology Department, Hospices Civils de Lyon, University Hospital of Lyon, Lyon, France

## Abstract

Early-onset systemic lupus erythematosus (SLE) is frequently associated with a more severe phenotype and may be linked to monogenic causes in at least 10% of all juvenile SLE cases. Recent advances in immunogenetics have identified Mendelian variants linked to inborn errors of immunity, underlying SLE. Toll-like receptor 7 (TLR7), an endosomal RNA sensor, has emerged as a key contributor to lupus pathogenesis through aberrant activation. We report a novel P435S gain-of-function (GOF) variant in *TLR7* identified in a female patient presenting with early-onset SLE, recurrent infection, and neuroinflammatory features. Functional assays demonstrated the gain-of-function effect, confirming its pathogenicity and supporting its role in disease onset and progression. To further define the clinical spectrum of TLR7 GOF-associated disease, we conducted a systematic review of 11 additional reported cases, highlighting shared and divergent phenotypic features. These findings expand the understanding of TLR7-mediated autoimmunity and underscore the importance of genetic screening in early-onset SLE with atypical features.

## Teaser

This study identifies a novel TLR7 variant causing monogenic lupus, recurrent infections, and neuroinflammation. The phenotype resembles type I interferonopathies, with neuroinflammation emerging as a core feature, with sustained interferon pathway activation.

## Graphical Abstract

**Figure:**
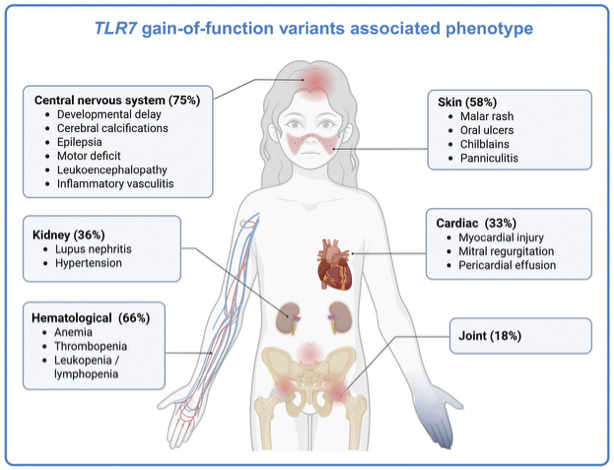

## Introduction

Systemic lupus erythematosus (SLE) is a prototypical systemic autoimmune disease characterized by chronic inflammation, severe multiorgan involvement, and the presence of antinuclear autoantibodies (1). Juvenile-onset SLE (jSLE), defined by disease onset before the age of 18 years, often follows a more severe clinical course, with increased prevalence of renal and neurological manifestations (2).

Over the last 15 years, advances in high-throughput sequencing technologies have uncovered monogenic forms of SLE particularly in familial or in early-onset cases (3, 4). Approximately 7–10% of jSLE cases are now attributed to inborn errors of immunity (4).

Toll-like receptor 7 (TLR7), encoded on the X chromosome, is a pattern recognition receptor expressed in endosomal compartments of immune cells, mainly in B cells and plasmacytoid dendritic cells. It plays a central role in antiviral defense by recognizing single-stranded RNA and initiating downstream signaling via MyD88, IRAK1/4, and TRAF6. These pathways activate NF-κB and IRF7 transcription factors, leading to the production of proinflammatory cytokines including type I interferons. Hemizygous loss-of-function mutations in *TLR7* have been implicated in severe COVID-19, underscoring its critical role in antiviral immunity (5).

Conversely, increased TLR7 dosage or gain-of-function (GOF) variants have been associated with heightened susceptibility to autoimmunity. A 2010 study in a Mexican cohort first linked *TLR7* copy-number variation to childhood-onset SLE (6). In 2022, the first identification and characterization of *TLR7* GOF were done in humans with subsequent reports showing that *TLR7*-GOF disease is associated with early-onset lupus and neuroinflammatory features (7, 8, 9, 10, 11). Additionally, GOF mutations in *UNC93B1*, a chaperone protein essential for TLR trafficking to endolysosomes, have been implicated in similar phenotypes (12, 13, 14, 15).

In this report, we describe a novel de novo heterozygous *TLR7* GOF variant in a young girl presenting with early-onset SLE, recurrent infections, and neuroinflammation. We also present a systematic review of previously reported cases to delineate the clinical and immunological spectrum of *TLR7* GOF-associated diseases.

## Results

### Case report

**Figure 1.**
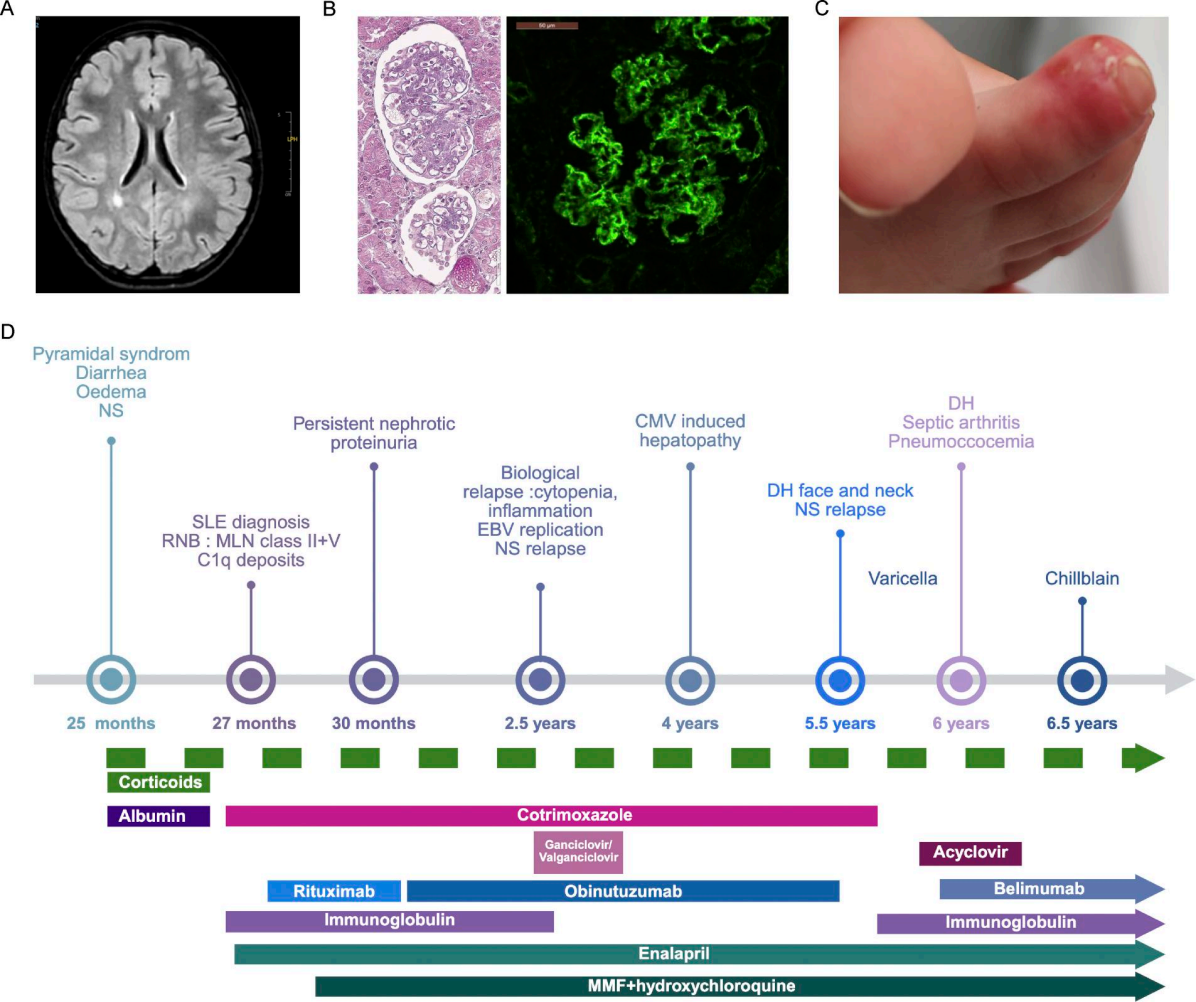
**Clinical phenotype of the proband. (A)** Brain MRI showing FLAIR hyperintensities in the deep periventricular white matter. **(B)** Kidney biopsy showing V and II class glomerulonephritis with C1q deposition. **(C)** Chilblain lesions. **(D)** Timeline of treatments and clinical signs. DH, dermohypodermitis; MLN, membranous lupus nephritis; MMF, mycophenolate mofetil; NS, nephrotic syndrome; RNB, renal biopsy; SLE, systemic lupus erythematosus. Created with BioRender (https://BioRender.com).

The proband is an 8-year-old female, born to nonconsanguineous parents of Algerian ancestry. She was born at 38 wk and 6 days of gestation, with a birth weight of 3,190 g and Apgar scores of 10 and 10. Early developmental milestones were delayed. She attained independent ambulation at 28 mo and exhibited persistent speech delay, being able to articulate six to seven words by the age of two and a half years. Subsequent developmental acquisitions were progressive and continuous. Neurological examination revealed a pyramidal syndrome predominantly affecting the lower limbs, resulting in a spastic gait. Brain and spinal magnetic resonance imaging (MRI) revealed subtle abnormalities, including FLAIR hyperintensities in the deep periventricular white matter (Fig. 1 A) with no calcification on the computed tomography (CT) scan.

At 25 mo of age, she presented with gastrointestinal features: melena, diarrhea, generalized edema, alongside nephrotic-range proteinuria. A kidney biopsy demonstrated class V (membranoproliferative) and class II (mesangioproliferative) glomerulonephritis with C1q deposition (Fig. 1 B). She also developed chilblains during winter seasons (Fig. 1 C).

At the age of three and a half, she developed bicytopenia, hypocomplementemia, and type III cryoglobulinemia, with positive anti-C1q antibodies, antinuclear antibodies 1/160, and negative anti-double-stranded DNA antibodies. These findings supported the diagnosis of SLE.

She was treated by a regimen of hydroxychloroquine, steroids, mycophenolate mofetil, further completed with sequential anti-B cell therapies (rituximab, obinutuzumab, and ongoing belimumab) due to refractory proteinuria and systemic manifestations (Fig. 1 D). She subsequently developed hypogammaglobulinemia, requiring intravenous immunoglobulin replacement. Despite supplementation, she experienced recurrent infections, including septic arthritis, dermohypodermitis, pneumococcal bacteremia, severe varicella, and cytomegalovirus hepatitis. She also had episodes of fluctuating hepatic cytolysis, attributed to CMV reactivation and lupus activity. At last follow-up, she remained clinically and biologically stable, with SLE features in remission, and residual mild pyramidal syndrome. She is behind in primary school and repeated first grade. She continues to have difficulties with oral expression. The most recent MRI, performed at 8 years of age, showed bilateral FLAIR hyperintensities in the deep parietal white matter, unchanged from previous imaging, suggestive of areas of delayed myelination. Neurological examination revealed a pyramidal syndrome of the lower limbs with widespread reflexes, limitation of ankle dorsiflexion, bilateral clonic tremor observed on this assessment, and bilateral Babinski signs.

### Genetic analysis

**Figure 2.**
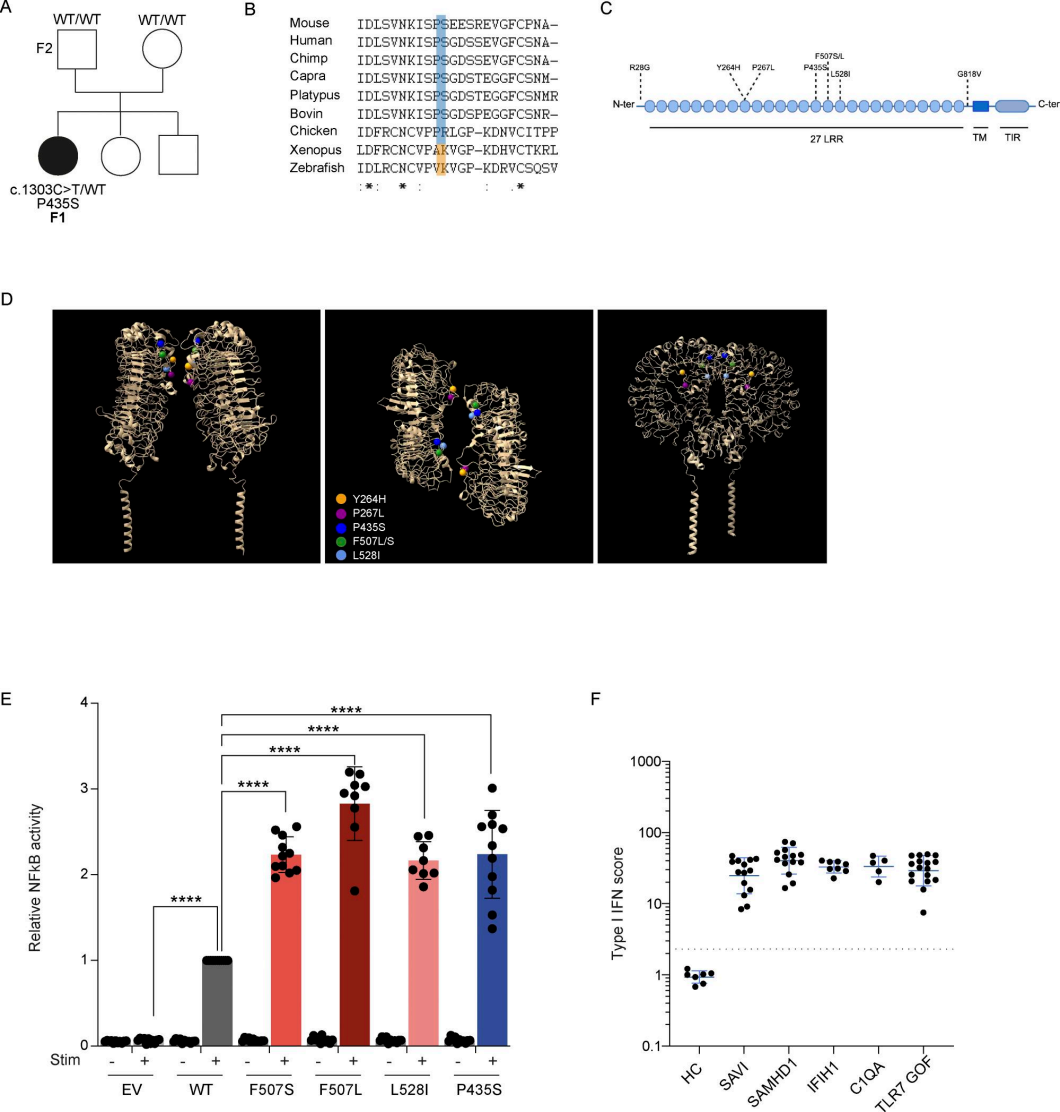
**Genetic analysis of the proband. (A)** De novo* TLR7* mutation identified by trio-based genome sequencing. **(B)** Conservation of the affected proline residue across species. **(C and D)** Location of P435S within the extracellular domain, relative to previously reported pathogenic variants (F507S/L, P267L, Y264H). **(E)** NF-κB reporter assay in HEK293T cells following R848 stimulation. **(F)** Sustained type I interferon signaling in the TLR7-GOF patient compared to patients with monogenic type I interferonopathies. HC, healthy controls; SAVI, STING-associated vasculopathy with onset in infancy; TIR, Toll/interleukin-1 receptor/resistance protein; TM, transmembrane; ****P < 0.0001.

Given the early-onset SLE and prominent neurological involvement, trio-based genome sequencing was performed through the France Genomic Medicine Plan, following an initial inconclusive solo exome analysis. This revealed a de novo heterozygous variant in the *TLR7* gene (NM_016562.4:c.1303C>T; p.(Pro435Ser)), hereafter referred to as P435S (Fig. 2 A). This variant was absent from the general population database gnomAD V4.1.0 and has not been previously reported in the literature. In silico predictions were inconclusive, showing discordant results: AlphaMissense classified the missense as ambiguous (0.412), rank exome variant ensemble learner (REVEL) predicted it as likely benign (0.128), and combined annotation dependent depletion (CADD) Phred assigned it a moderate score (20.30). The affected proline residue is highly conserved across species (Fig. 2 B) and located within the extracellular domain, though in proximity to previously reported pathogenic variants (e.g., F507S/L, P267L, Y264H) (Fig. 2, C and D) (16, 17). As this missense variant has not been previously reported, functional validation was warranted.

### Functional characterization

To this end, we overexpressed the wild-type (WT) *TLR7*, the previously reported F507S GOF variant, and the novel P435S variant in HEK293T cells. Using an NF-κB luciferase reporter assay, we measured the transcriptional activity following stimulation with the TLR7/8 agonist R848. The P435S variant exhibited enhanced sensitivity to R848, comparable to the F507S GOF control, while showing no spontaneous activation in the absence of stimulation (Fig. 2 E). The patient exhibited in vivo a sustained activation of type I interferon signaling, consistent with known monogenic type I interferonopathies, as indicated by a mean positive IFN score of 32 (17 measurements ranging from 7.5 to 49.5; normal range <2.3). This profile was comparable to that observed during the follow-up of four patients with monogenic type I interferonopathies (carrying *STING1* [*n* = 1], *SAMHD1* [*n* = 1], *IFIH1* [*n* = 1], or a *C1QA* [*n* = 1] variant) (Fig. 2 F).

These results support the pathogenicity of the P435S variant and its causal role in patient’s SLE phenotype.

### Systematic review of *TLR7* GOF cases

To better characterize the clinical spectrum associated with *TLR7* GOF variants, we conducted a systematic review of all 11 previously reported cases. Patients’ characteristics are summarized in Table 1.

**Table 1.** Clinical features of patients carrying TLR7 GOF variations

Reference	TLR7 variant	Inheritance	Sex, age at onset	Phenotype	Auto-Ab IFN signature	Evolution under treatment
(7) Family C	R28G	Het. Unknown	F, 18 yo	SLE with malar rash, arthralgia, Raynaud’s phenomenon, alopecia, fever, and oral ulcers, lymphopenia, thrombocytopenia	ANA+, anti-dsDNA+, anti-U1RNP+, SSA Ro52+, and Ro60+	Active episode (recurrent fever, headache, leucopenia) treated with corticosteroid and GM-CSF
(7) Family A	Y264H	Het. De novo	F, 7 yo	SLE with thrombocytopenia, arthralgia, and renal involvement (hypertensive crisis), mild mitral insufficiency and hemichorea	ANA+, +IFN signature	Severe thrombocytopenia (8G/L) that required corticosteroid, azathioprine, MMF, rituximab, IVIg, etanercept. Intermittent episode of chorea treated with haloperidol
(7) Family B (mother)	F507L	Het. Unknown	F, 25 yo	SLE and hemiplegic cerebral palsy of unclear etiology	NA	NA
(7) Family B (proband)	F507L	Het. Maternal	F, 9 yo	Relapsing NMO and transverse myelitis	ANA+, AQP4+	NMO exacerbations treated with IVIg and MMF. Received rituximab, then inebilizumab-cdon
(9)	P267L	Het. De novo	F, 13 mo	Anti-NMDAR encephalitis, SLE with pericardial effusion and profound hemolytic anemia, severe neuroinflammatory vasculitis with sudden-onset status epilepticus	ANA+, anti-dsDNA+ IFN signature+ (high)	Neurovasculitis treated initially with corticosteroid pulse IVIg, ofatumumab, cyclophosphamide, then baricitinib 2 months before HSCT. Currently only experiencing occasional hemolysis
(8) AGS571 (proband)	F507S	Het. Maternal	F, 4 yo	SLE with malar rash, anemia, lymphopenia, lupus nephritis class III/IV, intestinal ischemia, myocardial infarction, cerebral vasculitis, cerebral calcifications	ANA+, anti-dsDNA, aCL+, anti-β2GPI+, lupus anticoagulant IFN signature+(high, 14)	Died of acute myocardial infarction at the age of 17
(8) AGS571 (brother)	F507S	Hemizygous Maternal	M, 3 mo	Neonatal refractory epilepsy, dystonia, severe developmental delay, cerebral calcifications, leucopenia, and malar rash	ANA– IFN signature+(high)	Received corticosteroids, MMF, ruxolitinib. Currently, persistence of severe developmental delay and dystonia
(8) AGS571 (mother)	F507S	Unknown	F, 12 yo	Malar rash, thrombocytopenia, anemia	ANA+ IFN signature+(high)	NA
(8) AGS3740	L528I	Het. De novo	F, 1 yo	Refractory Evans-like syndrome, panniculitis, mild motor developmental delay, stereotypic movement disorder, leukoencephalopathy with brain atrophy and calcifications	ANA+, anti-SSA+	HSCT complicated by a chronic cutaneous GvHD treated with ruxolitinib. Currently, persistence of a slight motor deficit and hypokinesia
(11)	G818V	Het. De novo	F, 8 mo	Thrombocytopenia, anemia	ANA−	Relapse under rituximab, eltrombopag and baricitinib. HSCT. Currently no sign of disease activity
(10)	F506S	Somatic	M, 15 days	SLE with anemia, thrombocytopenia, rash, proteinuria, intestinal vasculitis, myocardial damage, thyroid dysfunction, intracranial calcification	ANA+	Bioclinical improvement under ruxolitinib 15 mg
This report	P435S	Het. De novo	F, 2 yo	SLE with lupus nephritis, thrombocytopenia, anemia, chilblains, and pyramidal syndrome	ANA+ Anti IFN+ IFN signature+ (high)	Clinically stable under belimumab with mild proteinuria and pyramidal syndrome

ANA, antinuclear antibodies; F, female; GM-CSF, granulocyte–macrophage colony-stimulating factor; GvHD, graft-versus-host disease; Het., heterozygous; HSCT, hematopoietic stem cell transplantation; IFN, interferon; IVIG: Intravenous Immunoglobulin; M, male; MMF, mycophenolate mofetil; NA, not available; NMDAR, N-methyl-D-aspartate receptor; NMO, neuromyelitis optica; SLE, systemic lupus erythematosus; SSA: Sjögren's Syndrome Antigen A; yo, years old.

The mean age at disease onset was 6.5 years, with a marked female predominance (10 out of 12 cases). Neuroinflammatory manifestations were the most frequently reported closely resembling those observed in type I interferonopathies. While penetrance was complete, clinical expressivity was variable—ranging from severe developmental delay and epilepsy to minimal or absent neurological symptoms.

Notably, signs of immunodeficiency were observed in several patients. One individual experienced recurrent invasive bacterial infection including dermohypodermitis, septic arthritis, septicemia—requiring immunoglobulin replacement therapy. Another patient presented with early-onset, recurrent viral infections.

Cardiovascular involvement was unexpectedly prominent in this population. One patient presented acute ischemia of a limb, one patient developed valvular heart disease, and two others exhibited myocardial damage, one of which was fatal.

Regarding therapies and outcome, six patients received anti-B cell therapies (rituximab, belimumab, ofatumumab, inebilizumab), often in combination with immunosuppressive agents such as steroids, azathioprine, mycophenolate mofetil with partial responses. Among five patients treated with JAK inhibitors (baricitinib, ruxolitinib), most showed clinical and biological improvement, although two relapses and one severe adverse effect were reported.

Three patients underwent hematopoietic stem cell transplantation (HSCT) due to refractory disease. HSCT led to sustained remission of autoimmune and hematologic manifestations, although neurological outcomes remained limited, with persistence of motor deficits and hypokinesia in one case. These observations suggest that while HSCT may offer a curative approach for systemic features, its efficacy in reversing neuroinflammation remains uncertain.

## Discussion

We report a novel lupus-causing *TLR7* GOF variant in a 2-year-old girl presenting with a multisystemic lupus phenotype, including neurological, renal, hematological, and cutaneous involvement.

The clinical presentation extends beyond classical SLE, with prominent neuroinflammatory features consistent with previous reports and reminiscent of type I interferonopathies, such as Aicardi–Goutières syndrome (AGS) (8). The neurological features are the most frequent manifestations in *TLR7* GOF disease, reported in 75% of patients and ranging from AGS-like syndrome to neuromyelitis optica with specific autoimmunity (anti-AQP4 antibodies), a rare phenotype also described in type I interferonopathy. This may reflect the sustained overexpression of type I interferon that appears similar to monogenic type I interferonopathies and higher than DNASE1L3 deficiency, a genetic defect associated with lupus phenotype and driven by a functional defect of extracellular DNA clearance (18). Contrary to *STING1*-associated vasculitis with onset in children or COPA syndrome, no patients carrying *TLR7* GOF displayed lung involvement.

Recurrent infections were noticed in some patients including the reported case. Enhanced TLR7 signaling might disrupt normal immune homeostasis, leading to defective immune responses despite an overall hyperinflammatory state and resulting in immune exhaustion. Since all patients were also treated with immunosuppressants, attributing infectious susceptibility to a single cause is challenging and requires further investigation. Cardiovascular involvement in TLR7 GOF patients was unexpectedly frequent and possibly fatal. It might result from immune dysregulation that drives microvascular inflammation, endothelial injury, and autoantibody-mediated cardiac damage. Furthermore, stimulation of TLR7 by the agonist R848 has been shown to accelerate lupus-associated cardiovascular pathology, including microvascular lesions and myocardial injury in lupus-prone mouse models (19).

The female predominance (10/12 reported cases) and the severe phenotype in the only male patient with germline mutation raise the possibility of sex-linked disease severity, potentially due to X-linked dosage effects or lethality in hemizygous males. The second male patient who exhibited mosaicism had a milder disease course, supporting this hypothesis.

Structural analysis of TLR7 reveals a clustering of pathogenic variants within the ligand-binding ectodomain. TLR7 is a 1049–amino acid protein, composed of 27 leucine-rich repeat (LRR) motifs, which form a curved solenoid structure essential for ligand recognition. Notably, the P435 residue lies within LRR15, which is outside the dimerization domain and adjacent to residues S434, G437, D438, and E441, known to contribute to the hydrophobic cavity binding single-stranded RNA and guanosine-based ligands (16). Previous studies have shown that the Y264H mutation enhances ligand affinity by stabilizing electrostatic interactions and increasing accessibility of the binding pocket (7). G818V variant can induce NF-κB/AP-1 activation in the absence of ligand suggesting a constitutive TLR7 activity (11).

Our patient’s clinical improvement and sustained remission under B cell–targeted therapies (belimumab, rituximab, and obinutuzumab) underscore the central role of B cells in TLR7-driven autoimmunity. This is supported by murine models, where enhanced TLR7 signaling promotes the survival and expansion of autoreactive B cells, particularly CD11c^+^ age-associated B cells (ABC, DN2), via extrafollicular pathways (7) and sustained type I IFN production (20). While rituximab achieved only partial B cell reduction in our patient, obinutuzumab led to a complete depletion, consistent with recent phase III data demonstrating superior efficacy of obinutuzumab in SLE (21). In *TLR7* GOF-associated disease, molecules capable of modulating TLR7 signaling and TLR7-driven autoimmunity represent promising therapeutic strategy for SLE. A phase 2 clinical trial is currently evaluating a selective TLR7/TLR8 antagonist (22). Beyond direct TLR7 blockade, modulation of downstream effectors such as STAT1 (23), IRAK4, and IRF5 (24, 25) may offer additional therapeutic avenues. Furthermore, strategies aimed at reducing TLR7 “dwell time” in endolysosomes by modulating its interaction with UNC93B1 or αvβ3 integrins are emerging as innovative approaches (26, 27, 28, 29, 30, 31, 32).

Collectively, these insights highlight the expanding spectrum of TLR7 GOF-associated disease, and underscore the need for precision medicine approaches tailored to the underlying molecular pathology.

## Materials and methods

### Patient

Ethical approval was obtained from the French Ethics Committee of CPP-SUD-Est III (2013). This study was registered under the EudraCT number (2012-A01449-34) and NCT01992666.

### Genetic study

Whole-genome sequencing was performed following the recommendations of France Genomic Medicine Plan. Whole blood–extracted genomic DNA was sequenced according to standard procedures for NovaSeq series sequencing instrument (Illumina). Sequencing data were aligned to the GRCh38p13 full assembly using bwa 0.7+. Variants were called by several algorithms including GATK4+, Bcftools1.10+, Manta1.6+, CNVnator0.4+, and annotated using the variant effect predictor. Detected variants were prioritized using in-house procedures. Further details are available on request.

### Immunofluorescence analysis

Human kidney biopsy specimens were fixed in formalin and embedded in paraffin for histological analysis. Sections were stained using periodic acid–Schiff to evaluate glomerular and interstitial morphology. For immunofluorescence studies, frozen sections were prepared to assess immune complex deposition, including C1q.

### 3D structure

3D structure visualization was performed using ChimeraX-Daily software.

### Cell lines and plasmids

A HEK293T cell line was cultured in a DMEM, high glucose, GlutaMAX supplement (Thermo Fisher Scientific) supplemented with 10% (vol/vol) heat-inactivated fetal calf serum (Dutscher), 10 mM HEPES, pH 7.5 (Thermo Fisher Scientific), and 0.04 mg/ml gentamicin (Thermo Fisher Scientific), and incubated at 37°C in 5%CO_2_. pCMV6 vector encoding WT TLR7 was previously described (5) and used as the parental vector for mutagenesis. The mutant P435S plasmid of TLR7 was generated via site-directed mutagenesis using the Q5 kit (E0554S; New England Biolabs) according to the manufacturer’s instructions (oligonucleotides used for mutagenesis: F: 5′-AGG​AGA​TTC​AAG​TGA​AGT​TGG​CTT​CTG​CTC​AAA​TGC​CAG​AAC-3′; R: 5′-GAA​GAT​GAT​ATT​TTA​TTC​ACT​GAA​AGA​TCT​ATG​ACT​TTC​AGT​CTT​TTA​AAT​TG-3′). cDNA sequence was confirmed by Sanger sequencing (Microsynth). Other mutants (F507S, F507L, and L528I) were previously described (8).

### Luciferase reporter assay

HEK293T cells were dispensed into a 6-well cell culture plate (1 × 10^6^ cells/well), left to adhere for 6 h, and transiently transfected using JET-PEI (Kit JetPEI Polyplus Transfection) according to the manufacturer’s instructions. Cells were transfected with indicated TLR7 constructs expressed in pCMV6 expression vector together with a pcDNA3.1 vector encoding human UNC93B1, a firefly luciferase reporter plasmid under the control of the NF-κB promoter (pGL4.32; Promega), and a constitutively expressed Renilla luciferase vector used for normalization (prL-SV40; Promega). 24 h after transfection, cells were detached, dispensed in a 96-well plate (50,000 cells/well), and left to adhere for 6 h. Transfected cells were then stimulated or not with the TLR7/8 ligand R848 (InvivoGen) at 100 ng/ml for 24 h. Luciferase activity was then measured using the Dual-Glo assay (Promega), with firefly luciferase signals normalized to Renilla luciferase activity. All transfection experiments were performed in triplicate. Data are presented as the percentage of luciferase activity obtained in the empty vector (EV) condition. Luminescence was measured on a TRISTAR 3 multimode Berthold microplate reader (Berthold Technologies GmbH & Co.KG). Data are expressed as fold induction, relative to unstimulated cells.

### IFN score

The expression of six interferon-stimulated genes (ISGs)—SIGLEC1 (sialic acid–binding Ig-like lectin 1), IFI27 (interferon α–inducible protein 27), IFI44L (interferon-induced protein 44-like), IFIT1 (interferon-induced protein with tetratricopeptide repeats 1), ISG15 (interferon-stimulated gene 15), and RSAD2 (radical S-adenosyl methionine domain containing 2)—was measured using a NanoString-based protocol (33). The absolute counts obtained for the six ISGs were normalized by the geometric mean of three housekeeping genes’ count number (β-actin, hypoxanthine phosphoribosyltransferase 1, and RNA polymerase II subunit A). The relative expression for each normalized ISG was calculated by dividing its value by the median normalized expression of the same ISG in a control group of 34 healthy volunteers. Finally, the median of these six ISGs’ relative expression was used to calculate the ISG expression score.

## Ethical statement

Written informed consent was obtained from the patient for publication of the case and accompanying images.

## Data Availability

Raw data underlying main figures are available from the corresponding author upon request.
